# Effect of a polyphenol-rich dietary pattern on subjects aged ≥ 60 years with higher levels of inflammatory markers: insights into microbiome and metabolome

**DOI:** 10.20517/mrr.2025.33

**Published:** 2025-10-21

**Authors:** Giorgio Gargari, Tomas Meroño, Gregorio Peron, Cristian Del Bo’, Mirko Marino, Antonio Cherubini, Cristina Andres-Lacueva, Paul Antony Kroon, Patrizia Riso, Simone Guglielmetti

**Affiliations:** ^1^Department of Food, Environmental and Nutritional Sciences (DeFENS), Universita Degli Studi di Milano, Milan 20133, Italy.; ^2^Biomarkers and Nutrimetabolomics Laboratory, Department de Nutrició, Ciències de l’Alimentació i Gastronomia, Institut de Recerca en Nutrició i Seguretat Alimentària (INSA), Facultat de Farmàcia i Ciències de l’Alimentació, Universitat de Barcelona (UB), Barcelona 08028, Spain.; ^3^Centro de Investigación Biomédica en Red de Fragilidad y Envejecimiento Saludable (CIBERFES), Instituto de Salud Carlos III, Madrid 28029, Spain.; ^4^Department of Molecular and Translational Medicine, University of Brescia, Brescia 25121, Italy.; ^5^Department of Geriatric Pathways of Frailty, Continuity of Care and Rehabilitation, Istituto di Ricovero e Cura a Carattere Scientifico - Istituto Nazionale di Ricovero e Cura per Anziani (IRCCS INRCA), Ancona 60127, Italy.; ^6^Department of Clinical and Molecular Sciences, Università Politecnica delle Marche, Ancona 60131, Italy.; ^7^Quadram Institute Bioscience, Norwich Research Park, Norwich NR4 7UQ, UK.; ^8^μbEat lab, Department of Biotechnology and Biosciences (BtBs), University of Milano-Bicocca, Milan 20126, Italy.

**Keywords:** Microbiome, metabolomics, inflammaging, intestinal permeability, polyphenols, healthy aging

## Abstract

**Background:** Aging may be associated with low-grade chronic inflammation (“inflammaging”) and gut microbiome alterations. Dietary polyphenols have been proposed as modulators of these processes. This study aimed to explore the effects of a polyphenol-rich diet (PR-diet) on inflammatory markers, gut microbiota, and metabolomic profiles in subjects aged ≥ 60 years stratified by baseline inflammation levels.

**Methods:** In this post-hoc analysis of the MaPLE (Microbiome mAnipulation through Polyphenols for managing Leakiness in the Elderly) randomized crossover trial, 50 subjects aged ≥ 60 years were categorized into two subgroups: high inflammation (cH) and low inflammation (cL). Participants received a PR-diet or a control diet for 8 weeks, with a washout period in between. Fecal, blood, and urine samples were analyzed using shallow shotgun metagenomics and untargeted metabolomics.

**Results:** The PR-diet was associated with a significant reduction in key inflammatory markers [e.g., interleukin-6 (IL-6), C-reactive protein] in the cH group. Distinct microbial shifts were observed, including an increase in *Blautia* and *Dorea* and a modest improvement in microbial diversity in cH subjects. Metabolomic analysis revealed group-specific changes, notably in polyphenol-derived metabolites.

**Conclusion:** These findings suggest that PR-diets may beneficially modulate inflammation and the gut microbial ecosystem in subjects aged ≥ 60 years with elevated baseline inflammation. Stratification by inflammatory status may improve the targeting and personalization of dietary interventions to support healthy aging.

## INTRODUCTION

The gut microbiome (GM), a pivotal player in inflammaging development, directly influences the immune system, modulating its response. Simultaneously, the immune system plays a crucial role in maintaining a stable host-microbe relationship^[1]^. Notably, pathological conditions such as acute diarrheal disease, inflammatory bowel disease (IBD), liver disease, and colon-rectal cancer have been linked to reduced GM biodiversity with increased proportions of facultative anaerobes. These diseases are characterized by alterations in immune system functionality, which can be exacerbated by the establishment of GM with reduced taxonomic richness^[2]^. Changes in bacterial metabolic flux, leading to diminished production of essential metabolites, and the concurrent attenuation of host defenses against pathogen invasion represent further consequences of a decline in microbial biodiversity within the GM^[3]^.

Subjects aged ≥ 60 years undergo well-documented shifts in gut microbiota composition. These changes particularly affect beneficial taxa such as *Bifidobacterium*, *Lactobacillus*, *Faecalibacterium*, and members of the *Lachnospiraceae* family, while promoting an increase in opportunistic or pro-inflammatory pathobionts, including *Enterobacteriaceae* and other *Pseudomonadota*. Such alterations are associated with decreased production of short-chain fatty acids, increased intestinal permeability (IP), and the onset of systemic low-grade inflammation, which are hallmarks of the process known as “inflammaging”. These microbiome shifts may contribute to frailty, immune dysfunction, and the development of age-related diseases, making adults aged ≥ 60 years a particularly relevant target for dietary strategies aimed at modulating the gut microbiota. Furthermore, during aging, the GM undergoes a general reduction in microbial diversity, which was proposed as a biomarker of an altered GM, bearing age-dependent implications for health^[3]^. In particular, the GM also appears to be relevant for inflammaging, a prevalent condition among adults aged ≥ 60 years that is characterized by chronic, low-grade inflammation intricately connected to the onset of age-related diseases, posing a substantial risk for morbidity and mortality^[4]^. Diet may influence inflammaging both directly, by regulating the immune system, and indirectly, by modulating the GM. For instance, various dietary compounds, such as epigallocatechin-3-gallate (found in green tea), phenethyl isothiocyanate (found in cruciferous vegetables), and parthenolide (extracted from the plant feverfew), can modulate the activation of Toll-like receptors that have important roles in the induction of innate immune responses for host defense against invading microbial pathogens^[5-7]^. Furthermore, molecules derived from the diet may be fermented in the gut into metabolites that can influence host metabolism, such as butyrate, trimethylamine, p-cresol, and hydrogen sulfide^[8-11]^.

A large proportion of polyphenols, plant-derived compounds with potential anti-inflammatory properties, reach the colon after ingestion and are fermented by colonic microbiota^[12]^. The resulting metabolites could exhibit various beneficial effects, such as inhibiting opportunistic pathogen growth, restoring intestinal mucosal barrier function, modulating immunological response, and reducing oxidative stress^[13]^. Polyphenols play a critical role in managing inflammation through multifaceted biochemical mechanisms, including the inhibition of key inflammatory pathways such as nuclear factor-kappa B (NF-κB) and the Nucleotide-binding oligomerization domain-like receptor family, pyrin domain containing 3 (NLRP3) inflammasome, modulation of mitogen-activated protein kinase (MAPK) signaling, and regulation of immune cell functions^[14,15]^. This extensive network of interactions underscores the potential of polyphenols as therapeutic agents in managing inflammation-related diseases. In summary, the consumption of polyphenol-rich foods emerges as a promising strategy to positively modulate gut microbiota composition and reduce systemic inflammation^[16]^. This could be of interest in specific populations, such as adults aged ≥ 60 years, who are more prone to an inflammatory status. In this context, we developed the project titled “*Gut and blood microbiomics for studying the effect of a polyphenol-rich dietary pattern on intestinal permeability in the elderly*” (Microbiome mAnipulation through Polyphenols for managing Leakiness in the Elderly (MaPLE))^[17]^, which was based on a dietary intervention trial where the effects of a polyphenol-rich diet (PR-diet) were compared with a control diet (CT-diet). This study provides evidence that a PR-diet may have a beneficial role in improving IP-associated conditions, particularly in adults aged ≥ 60 years. Marino *et al.* reported that such a diet could help manage IP-related conditions and identified calprotectin as a promising biomarker of intestinal and systemic inflammation^[18]^. Peron *et al.* highlighted the complex interactions between polyphenol consumption, IP, and gut microbiota composition, emphasizing the potential for personalized dietary interventions in adults aged ≥ 60 years^[19]^. Del Bo’ *et al.* demonstrated that a PR-diet significantly reduces serum zonulin levels, an indirect marker of IP, while also lowering blood pressure and promoting beneficial shifts in gut microbiota, particularly by increasing fiber-fermenting and butyrate-producing bacteria^[20]^. Lastly, Guglielmetti *et al.* confirmed the reliability of this dietary intervention, supporting its potential inclusion in future dietary guidelines for IP management in adults aged ≥ 60 years and at-risk populations^[17]^.

The present study was conducted as a continuation of the MaPLE trial to further investigate the complex relationship between diet, the GM, IP, and inflammation in aging, which remains not fully understood. By clustering subjects into two distinct groups based on their inflammation level, we were able to identify a robust association between inflammaging, intestinal microbiome, and metabolic changes. This research contributes to the existing body of knowledge by providing deeper insights into how PR-diets can modulate these factors, offering potential avenues for dietary interventions aimed at promoting healthy aging. Specifically, by examining changes in fecal, serum, and urine metabolites, alongside the analysis of IP, this study aims to uncover potential biomarkers and metabolic pathways associated with inflammaging. These findings may pave the way for personalized dietary strategies to manage age-related inflammation and gut dysfunction, providing significant implications for public health and aging populations.

## METHODS

### Trial design and participants

**Figure 1:**
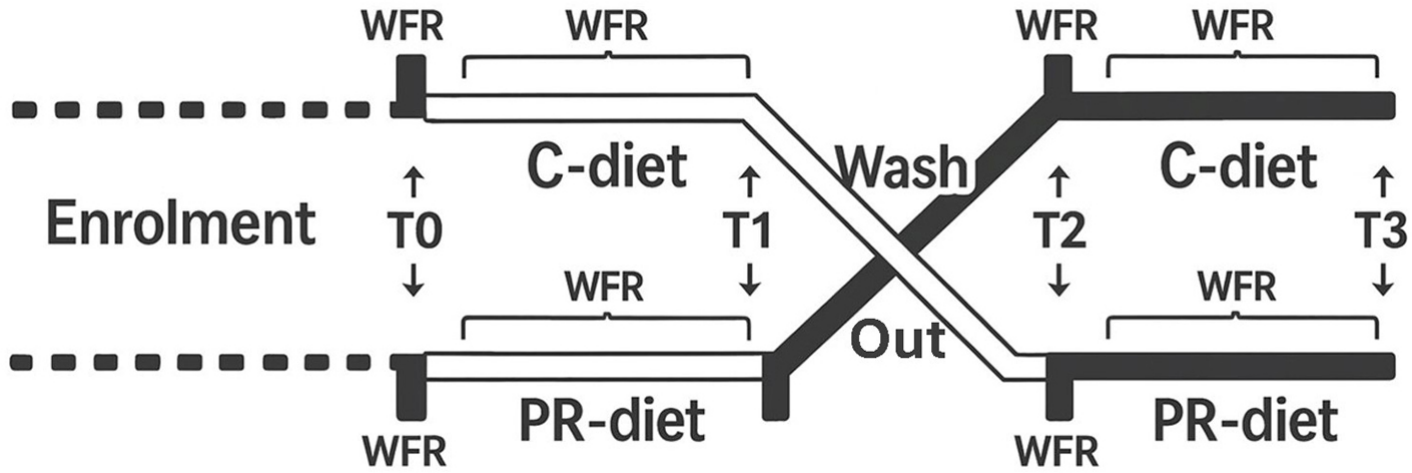
Schematic overview of the study protocol. WFR: Weighed food records; T0-T3: time points corresponding to intervention phases; C-diet: control diet; PR-diet: polyphenol-rich diet.

This study is a post-hoc analysis of samples collected during the project titled “*Microbiomics mAnipulation through Polyphenols for managing the Leakiness in the Elderly*” (MaPLE). The MaPLE dietary intervention trial has been previously described in detail^[17]^. Briefly, a total of 51 adults aged ≥ 60 years were enrolled and completed the intervention. However, 50 participants with complete clinical, microbiome, and metabolomic datasets were included in the present post-hoc analysis [Table 1]. The study consisted of an 8-week, randomized, controlled, crossover dietary intervention in which a group of individuals aged ≥ 60 years consumed a PR-diet and/or a CT-diet in a randomized sequence [Figure 1].

**Table 1:** Number of subjects per group included in the omics analysis

**Group**	**Allocated (*n*)**	**Dropout after 1st intervention**	**Completed 1st phase**	**Dropout after 2nd intervention**	**Completed both phases**	**Omics analysis**	**Total**	**Clustering**
PR → C	35	7	28	0	28	28	50	cH = 23
C → PR	31	5	26	3	23	22		cL = 27

Participants were stratified into two groups based on baseline CRP levels: higher inflammation (cH, *n* = 23) and lower inflammation (cL, *n* = 27). PR: Polyphenol-rich; C: control; CRP: C-reactive protein.

The PR-diet was specifically formulated to ensure a significantly higher intake of polyphenols compared to the CT-diet. It included a variety of polyphenol-rich foods such as blood orange and its juice, pomegranate juice, green tea, *Renetta Canada* apples and apple purée, dark chocolate, and mixed berries (e.g., blueberries, blackberries, raspberries), along with derived products. These items were selected for their high content of flavonoids, phenolic acids, and stilbenes. Based on quantification using the Folin-Ciocalteu assay^[20]^, the average polyphenol intake during the PR-diet phase was approximately 1,391 mg/day.

In contrast, the CT-diet was isoenergetic and matched in macronutrient composition, but it deliberately excluded the above-mentioned polyphenol-rich foods. As a result, the total polyphenol content of the CT-diet was considerably lower, averaging approximately 812 mg/day. This design enabled a controlled comparison of the effects of polyphenol intake while minimizing confounding factors related to differences in energy or macronutrient distribution.

Blood, feces, and urine samples were collected at the beginning and the end of each phase of the intervention. A sample size of 50 adults aged ≥ 60 years was determined to be sufficient to detect an IP reduction of 30% with 80% power at a 0.05 significance level, accounting for a 15% dropout rate^[17]^. Based on this calculation, subjects were enrolled and selected by physicians at the Civitas Vitae nursing home (Padua, Italy) according to specific inclusion and exclusion criteria^[17]^. Subjects included in the study were ≥ 60 years of age, had generally good nutritional and cognitive status, and exhibited increased IP, as indicated by elevated serum zonulin levels. All participants were informed about the study protocol and signed informed consent before enrolment. The study protocol was approved by the Ethics Committee of the University of Milan (Ref.: 6/16/CE_15.02.16_Verbale_All-7). The trial was registered in the ISRCTN public registry (ISRCTN10214981). Of the 66 subjects initially enrolled, 51 completed the trial.

### Blood sampling and biomarker analysis

After an overnight fast, blood samples were collected into tubes containing silica gel for serum separation. Tubes were kept at room temperature for at least 30 min before centrifugation at 1,400 g and 4 °C for 15 min to obtain serum. The serum was aliquoted into labeled vials and stored at -80 °C until analysis. Physiological parameters were measured as previously described^[17]^. Serum zonulin levels were quantified using the Immunodiagnostik^®^ ELISA kit (Bensheim, Germany), following the established protocol^[17]^. Serum and urine metabolites were processed and measured as previously described^[19,21]^. Inflammatory markers, including C-reactive protein (CRP), tumor necrosis factor-alpha (TNF-α), and interleukin-6 (IL-6), were quantified in serum samples collected at the beginning and end of each intervention period using specific Quantikine ELISA kits. Additional immune activation markers, serum calprotectin and fecal calprotectin, were measured using the Hycult Biotech ELISA kit (Pennsylvania, USA).

### DNA Extraction and library preparation

Fecal samples were stored at −80 °C prior to assessment of the intestinal microbiome via shallow shotgun sequencing. DNA was extracted using the QIAGEN DNeasy PowerSoil Pro Kit, following the manufacturer’s instructions. DNA concentrations were measured using a Qubit 4 fluorometer with the Qubit™ dsDNA HS Assay Kit (Thermo Fisher Scientific).

DNA libraries were prepared using the Nextera XT DNA Library Preparation Kit (Illumina) and Nextera Index Kit (Illumina), with 1 ng total DNA input. Genomic DNA was fragmented using the Illumina Nextera XT fragmentation enzyme in proportion to the input DNA. Combinatorial dual indexes were added to each sample, followed by 12 polymerase chain reaction (PCR) cycles to construct the libraries. Libraries were purified with AMpure magnetic beads (Beckman Coulter) and eluted in QIAGEN EB buffer. DNA concentrations were quantified using the Qubit 4 fluorometer and Qubit™ dsDNA HS Assay Kit. Sequencing was performed on an Illumina HiSeq X platform (2x150bp). The resulting FASTQ files have been deposited in the European Nucleotide Archive (ENA) of the European Bioinformatics Institute under accession code PRJEB76236.

### Bioinformatics analysis

Raw sequencing reads obtained from the Illumina HiSeq X platform totaled 150,728,010 (mean: 742,503; median: 665,186). After quality control, the number of reads decreased to 149,135,812 (mean: 734,659; median: 658,103). Quality-filtered reads were processed using the bioBakery pipeline^[22]^, starting with cleaning by KneadData, a tool designed for quality control and filtering of sequencing reads. From the filtered sequences, paired-end reads (R1 and R2) were combined, and microbial community profiling was performed using MetaPhlAn 3 with the Chocophlan full database. Subsequently, the HMP Unified Metabolic Analysis Network v.3 (HUMAnN 3) was applied to characterize the metabolic potential of the microbial communities. Data were normalized to counts per million, following bioBakery recommendations.

### Targeted metabolomics analyses

Plasma and urine metabolomics analyses were conducted as described previously^[19,23]^. Briefly, targeted metabolomics was performed using authentic standards for metabolites derived from diet, gut microbiota fermentation, bioactive compounds, and endogenous sources^[24]^.

### Statistical analyses

Statistical analyses were conducted using R software (version 3.1.2). Of the volunteers who completed the MaPLE study (*n* = 51), data from 50 subjects with complete metabolome, microbiome, and inflammatory parameter datasets were analyzed. Subjects were clustered into two groups (cH and cL) using the PAM (Partitioning Around Medoids) algorithm based on the Jensen-Shannon distance matrix. The goodness of clustering was evaluated with the analysis of similarities (ANOSIM) statistic. Baseline differences between groups were assessed using unpaired Student’s *t*-tests or Mann-Whitney *U* tests, depending on the normal distribution of data as determined by the Shapiro-Francia normality test. To control for multiple comparisons, Benjamini-Hochberg false discovery rate (FDR) correction was applied separately to each dataset—microbiome (taxonomic relative abundances), metabolome (urinary and fecal metabolites), and physiological/inflammatory parameters. Adjusted *P*-values (q-values) ≤ 0.05 were considered statistically significant. This correction was consistently applied across analyses to control the expected false positive rate within each data domain. Significant effects of dietary intervention were assessed by repeated measures analysis of variance (ANOVA) or non-parametric ANOVA, depending on the normality of the data. Correlations at baseline were evaluated using Kendall correlation analysis. Significance thresholds were set at *P* ≤ 0.05, with trends noted for 0.05 ≤ *P* ≤ 0.1. Alpha diversity was assessed using Chao1, Shannon, and Simpson indexes, and beta diversity was evaluated by partial least squares discriminant analysis (PLSDA).

## RESULTS

### Inflammatory markers form two distinct clusters in individuals aged ≥ 60 years at baseline

**Figure 2:**
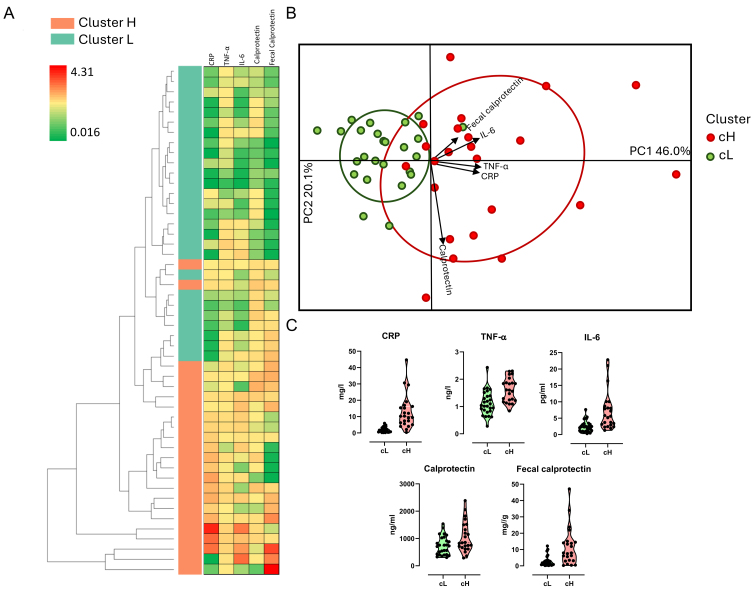
Clustering of subjects based on inflammatory markers. Stratification into two distinct clusters, namely cH (cluster high) and cL (cluster low), was based on levels of C-reactive protein (CRP), tumor necrosis factor-alpha (TNF-α), interleukin-6 (IL-6), serum calprotectin (Calprotectin), and fecal calprotectin. (A) Heatmap depicting an unsupervised model of inflammatory variables; high and low abundances are represented by red and green colors, respectively; (B) Biplot illustrating subjects according to inflammatory markers; (C) Violin plot displaying inflammatory marker levels grouped by cluster.

Serum (CRP, TNF-α, IL6, and calprotectin) and fecal (calprotectin) inflammatory markers were assessed in 50 volunteers aged ≥ 60 years participating in the MaPLE study. A heatmap with an accompanying hierarchical clustering tree was generated based on these markers, showing a clear separation among subjects, which was supported by ANOSIM results (R = 0.49; *P* = 0.01) [Figure 2A]. An unsupervised clustering algorithm further stratified participants into two distinct inflammatory profiles: a high inflammation group (cH; *n* = 23) and a low inflammation group (cL; *n* = 27) [Figure 2B]. The cH group exhibited higher median levels for all inflammatory markers compared to the cL group (CRP: 12.7 *vs.* 1.91 mg/L; TNF-α: 1.60 *vs.* 1.12 ng/L; IL6: 7.12 *vs.* 2.37 pg/L; serum calprotectin: 1,053 *vs.* 738 ng/mL; fecal calprotectin: 11,416.1 *vs.* 2,869.3 ng/g) [Figure 2C].

Other physiological parameters also differed significantly or showed significant trends between the two clusters. Specifically, subjects in the cH group had lower levels of zonulin (*P* = 0.036), total cholesterol (TC) (*P* = 0.088), and high-density lipoprotein (HDL) (*P* = 0.005) compared to those in the cL group. Conversely, the cL group exhibited lower TC/HDL (*P* = 0.038) and low-density lipoprotein (LDL)/HDL (*P* = 0.017) ratios, insulin (*P* = 0.1), Homeostasis Model Assessment (HOMA) index (*P* = 0.086), and soluble vascular cell adhesion molecule-1 (sVCAM, *P* = 0.002), CRP (*P* < 0.001), TNF-α (*P* < 0.001), IL-6 (*P* < 0.001), calprotectin (*P* = 0.048), and fecal calprotectin (*P* < 0.001) [Table 2].

**Table 2:** Differential analysis of physiological parameter levels

**Physiological markers**	**Normality (Shapiro-Francia test)**	***P*-value**	**Mean/Median cL**	**Mean/Median cH**
Zonulin	Yes	0.036	45.5	38.46
TC	Yes	0.088	202.48	178.48
HDL	Yes	0.005	52.67	40.87
TC-HDL ratio	Yes	0.038	3.98	4.58
LDL-HDL ratio	Yes	0.017	2.37	2.85
Insulin	No	0.1	5.9	7
HOMA index	No	0.086	1.4	1.8
sVCAM	No	0.002	678.8	1,192.4
CRP	No	< 0.001	1.6	10.2
TNF-α	Yes	< 0.001	1.13	1.6
IL-6	No	< 0.001	2.07	5.61
Calprotectin	No	0.048	727.6	841.3
Fecal calprotectin	No	< 0.001	1,824	7,930

A comprehensive differential analysis of physiological parameters between the two clusters (cL and cH) is presented, showing only parameters with a significance level of *P* ≤ 0.1. *P*-values were calculated using parametric (*t*-test) and non-parametric (Mann-Whitney *U*) tests, depending on normality assessed by the Shapiro-Francia test. Mean or median values are reported according to the test applied. Zonulin (ng/mL); TC (mg/dL); Insulin (μU/mL); sVCAM (ng/mL); CRP (mg/L); TNFα (pg/mL); IL-6 (pg/mL); Calprotectin (ng/mL); Fecal calprotectin (μg/g). cL: Lower inflammation; cH: higher inflammation; TC: total cholesterol; HDL: high-density lipoprotein; LDL: low-density lipoprotein; HOMA: Homeostasis Model Assessment; sVCAM: soluble vascular cell adhesion molecule-1; CRP: C-reactive protein; TNF-α: tumor necrosis factor-alpha; IL-6: interleukin-6.

### Fecal microbiome and metabolite diversity between the two clusters at baseline

**Figure 3:**
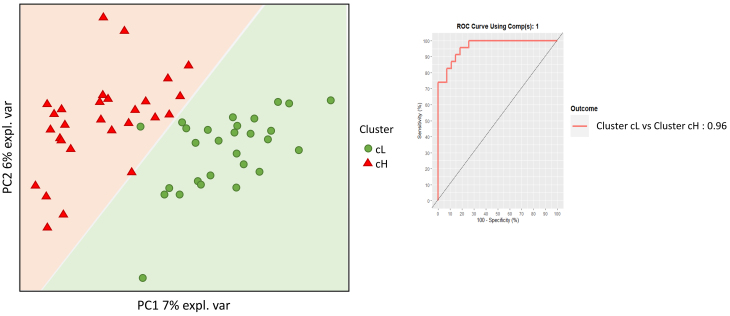
Supervised beta-diversity PLSDA. Microbiota analysis was performed using supervised beta-diversity PLSDA to distinguish between the cL and cH clusters. This analysis shows the arrangement of subjects according to their microbiota composition. The receiver operating characteristic (ROC) curve and the area under the curve (AUC) are presented to assess the microbiota’s discriminatory power between the two clusters. PLSDA: Partial least squares discriminant analysis; cL: lower inflammation; cH: higher inflammation.

**Figure 4:**
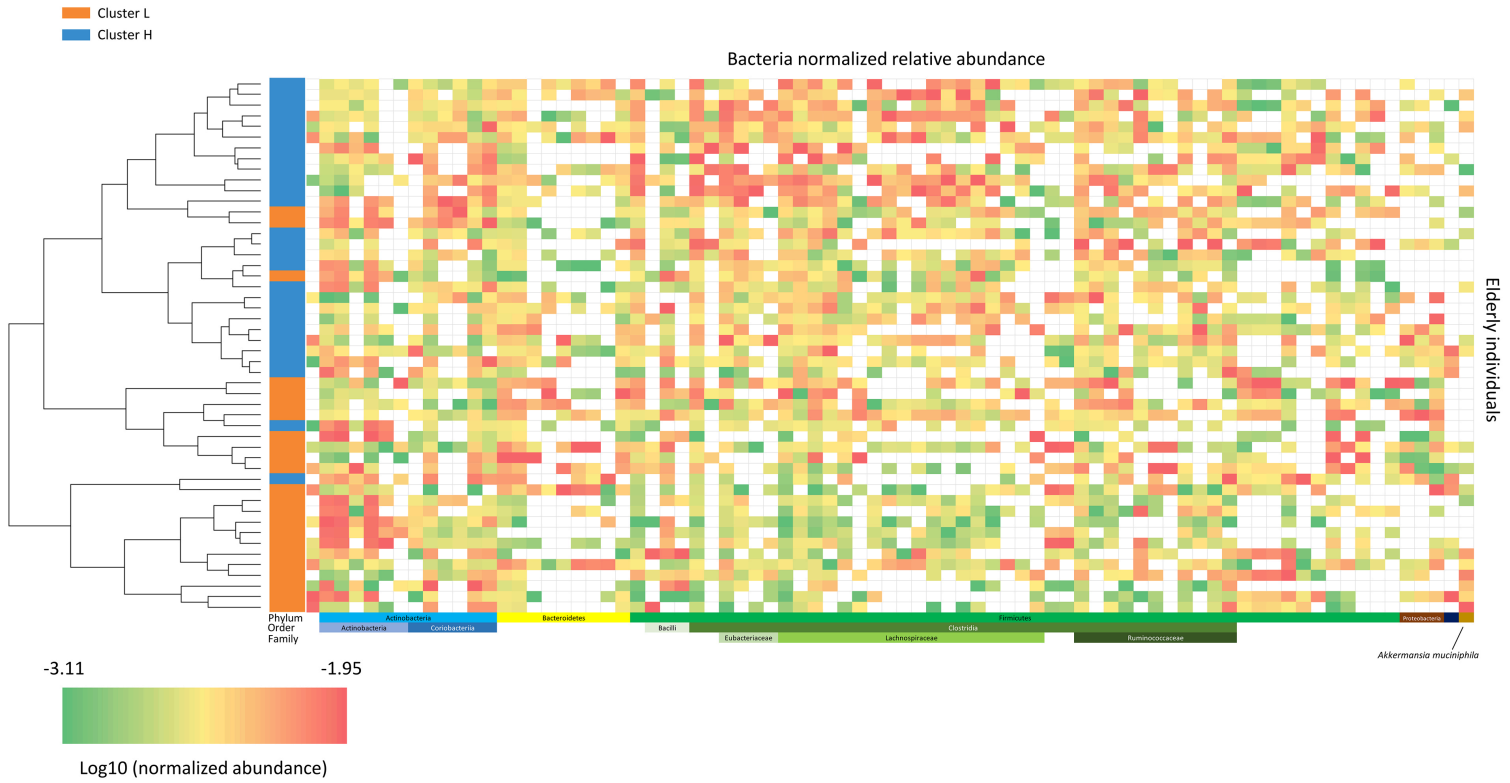
Microbiota heatmap. This heatmap illustrates the normalized relative abundance (log10 scale) of bacterial taxa for each subject. Lower bacterial levels are shown in green, and higher levels in red. The color-coded bar on the left indicates subjects’ cluster membership (cL or cH), arranged according to the hierarchical clustering tree. cL: Lower inflammation; cH: higher inflammation.

Fecal DNA from the 50 individuals aged ≥ 60 years was analyzed by shallow shotgun metagenomics, yielding a total of 1,196,970,158 raw reads, with an average of 5,896,404 reads per sample (Standard Deviation (SD): 1,530,289; median: 5,686,622; range: 2,824,144–10,994,558). Microbiome analysis focused on bacterial composition and utilized supervised beta diversity via partial least squares-discriminant analysis (PLS-DA), which robustly distinguished samples corresponding to the cH and cL groups [Figure 3]. This discrimination was confirmed by an unsupervised relational tree analysis [Figure 4]. Significant changes were observed at the phylum level: an increase in Bacillota (formerly Firmicutes) in the cH group was characterized by increased Lactobacillales and decreased Eubacteriales (formerly Clostridiales). The cH group also had lower levels of Bacteroidales and higher levels of Bifidobacteriales compared to cL [Table 3].

**Table 3:** Differential analysis of bacterial levels between the two clusters (cL and cH)

**Taxonomy**	***P*-value**	**Median**	
		**cH**	**cI**
***p_Actinobacteria;c_Actinobacteria;o_Bifidobacteriales;f_Bifidobacteriaceae;g_Bifidobacterium;s_Bifidobacterium_bifidum***	**0.036**	0	0.01
***p_Actinobacteria;c_Actinobacteria;o_Bifidobacteriales;f_Bifidobacteriaceae;g_Bifidobacterium;s_Bifidobacterium_dentium***	**0.029**	0	0.06
***p_Actinobacteria;c_Coriobacteriia;o_Eggerthellales;f_Eggerthellaceae;g_Gordonibacter;s_Gordonibacter_pamelaeae***	**0.042**	0.41	0.14
*p_Bacteroidetes;c_Bacteroidia;o_Bacteroidales;f_Bacteroidaceae;g_Bacteroides;s_Bacteroides_caccae*	0.075	0.02	0
*p_Bacteroidetes;c_Bacteroidia;o_Bacteroidales;f_Bacteroidaceae;g_Bacteroides;s_Bacteroides_vulgatus*	0.073	0.18	0
*p_Bacteroidetes;c_Bacteroidia;o_Bacteroidales;f_Barnesiellaceae;g_Barnesiella;s_Barnesiella_intestinihominis*	0.086	0.05	0
***p_Firmicutes***	**0.003**	54.68	35.49
***p_Firmicutes;c_Bacilli;o_Lactobacillales***	**0.029**	0.23	2.39
***p_Firmicutes;c_Bacilli;o_Lactobacillales;f_Lactobacillaceae;g_Lactobacillus***	**0.045**	0	0.08
*p_Firmicutes;c_Bacilli;o_Lactobacillales;f_Streptococcaceae*	0.07	0.21	1.12
*p_Firmicutes;c_Bacilli;o_Lactobacillales;f_Streptococcaceae;g_Streptococcus*	0.059	0.21	1.12
***p_Firmicutes;c_Bacilli;o_Lactobacillales;f_Streptococcaceae;g_Streptococcus;s_Streptococcus_thermophilus***	**0.044**	0	0.06
***p_Firmicutes;c_Clostridia;o_Clostridiales***	**0.001**	50.43	30.1
***p_Firmicutes;c_Clostridia;o_Clostridiales;f_Eubacteriaceae;g_Eubacterium***	**0.003**	3.07	0.92
***p_Firmicutes;c_Clostridia;o_Clostridiales;f_Eubacteriaceae;g_Eubacterium;s_Eubacterium_hallii***	**0.001**	0.77	0.15
***p_Firmicutes;c_Clostridia;o_Clostridiales;f_Lachnospiraceae***	**0.001**	21.93	7.07
***p_Firmicutes;c_Clostridia;o_Clostridiales;f_Lachnospiraceae;g_Anaerostipes;s_Anaerostipes_hadrus***	**< 0.001**	1	0.19
*p_Firmicutes;c_Clostridia;o_Clostridiales;f_Lachnospiraceae;g_Blautia*	0.098	3.06	1.66
*p_Firmicutes;c_Clostridia;o_Clostridiales;f_Lachnospiraceae;g_Blautia;s_Blautia_obeum*	0.051	1	0.59
*p_Firmicutes;c_Clostridia;o_Clostridiales;f_Lachnospiraceae;g_Blautia;s_Ruminococcus_torques*	0.072	0.45	0
*p_Firmicutes;c_Clostridia;o_Clostridiales;f_Lachnospiraceae;g_Coprococcus*	0.067	1.01	0.14
*p_Firmicutes;c_Clostridia;o_Clostridiales;f_Lachnospiraceae;g_Coprococcus;s_Coprococcus_eutactus*	0.098	0	0
***p_Firmicutes;c_Clostridia;o_Clostridiales;f_Lachnospiraceae;g_Dorea***	**0.011**	1.79	0.74
*p_Firmicutes;c_Clostridia;o_Clostridiales;f_Lachnospiraceae;g_Dorea;s_Dorea_formicigenerans*	0.085	0.44	0.14
*p_Firmicutes;c_Clostridia;o_Clostridiales;f_Lachnospiraceae;g_Dorea;s_Dorea_longicatena*	0.055	1.04	0.53
***p_Firmicutes;c_Clostridia;o_Clostridiales;f_Lachnospiraceae;g_Fusicatenibacter;s_Fusicatenibacter_saccharivorans***	**0.004**	3.34	0.32
***p_Firmicutes;c_Clostridia;o_Clostridiales;f_Ruminococcaceae***	**0.007**	16.59	9.73
***p_Firmicutes;c_Clostridia;o_Clostridiales;f_Ruminococcaceae;g_Ruminococcus***	**0.006**	9.24	1.79
***p_Firmicutes;c_Clostridia;o_Clostridiales;f_Ruminococcaceae;g_Ruminococcus;s_Ruminococcus_bromii***	**0.028**	7.15	1.7
***p_Firmicutes;c_Negativicutes;o_Veillonellales;f_Veillonellaceae;g_Megasphaera***	**0.036**	0	0.09

A comprehensive comparison of bacterial levels between clusters cL and cH is presented. Parameters with a significance level of *P* ≤ 0.1 are shown, with those reaching *P* < 0.05 highlighted in bold. *P*-values were calculated using non-parametric tests. Median values, expressed as normalized relative abundances, are reported for clarity. Taxonomic notation is as follows: p: Phylum; c: class; o: order; f: family; g: genus; s: species. cH: higher inflammation; cL: lower inflammation.

**Figure 5:**
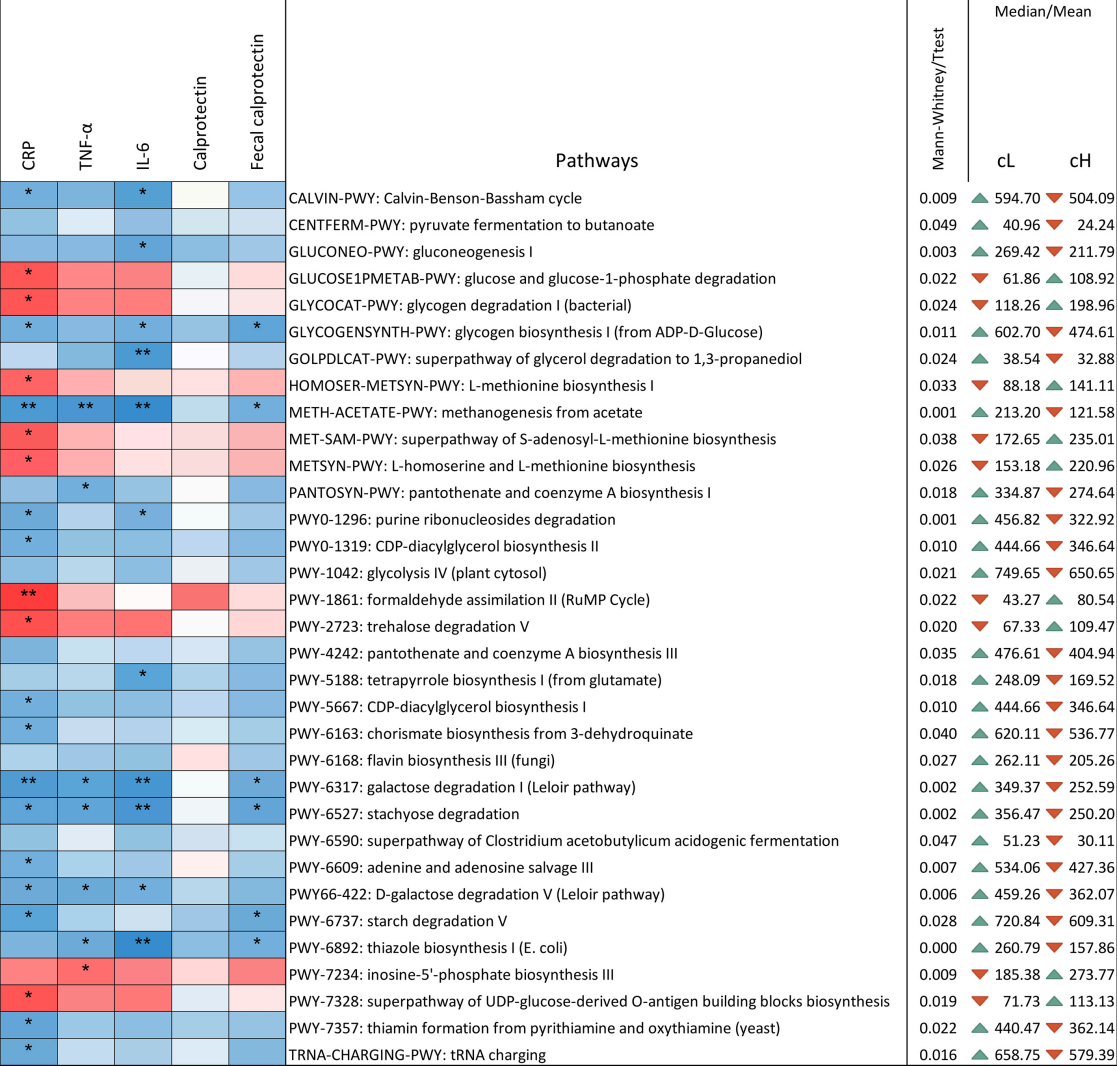
Correlation between pathways and inflammatory markers determined using the Kendall test. The ꞇ index defines the color scale (blue for negative correlation, red for positive correlation), and asterisks indicate significance levels: ^*^0.1 < *P* < 0.05; ^**^0.001 < *P* < 0.01; ^***^P < 0.001. Only pathways showing differential presence between cL and cH are displayed. *P*-values were computed using both parametric (*t*-test) and non-parametric (Mann-Whitney *U*) tests, according to the results of the Shapiro-Francia normality test. Mean or median values, depending on the applied test, are reported. The triangular indicator denotes whether the level is higher (green) or lower (red) in one cluster relative to the other. Values are normalized and expressed as copies per million for genes associated with each pathway. cL: Lower inflammation; cH: higher inflammation.

Pathway richness differed between clusters [Figure 5]. The pathways significantly reduced in cH were primarily associated with decreased abundance of the genus *Blautia* and the species *Anaerostipes hadrus*. In contrast, pathways increased in cH were mainly driven by *Bifidobacterium longum* and unclassified bacteria. Notably, the super-pathways of *Clostridium acetobutylicum* acidogenic fermentation and glycerol degradation to 1,3-propanediol decreased in cH, reflecting reduced levels of Clostridiales.

Out of 33 pathways differing significantly between clusters, 28 correlated with at least one inflammatory marker [Figure 5]. All pathways negatively correlated with inflammatory markers were lower in cH compared to cL, while those positively correlated were higher in cH. The remaining five pathways (pyruvate fermentation to butanoate, glycolysis IV, pantothenate and coenzyme A biosynthesis III, flavin biosynthesis III, and the superpathway of *Clostridium acetobutylicum* acidogenic fermentation) were decreased in cH but showed no correlation with inflammatory markers.

Metabolites in serum, urine, and feces from the 50 participants were quantified and compared between clusters. Significant differences are presented in Supplementary Table 1. Several metabolites linked to inflammation, such as 2-methylpyrogallol sulfate (2-MePyr-S), dihydroresveratrol (DHRSV-G2), asymmetric dimethylarginine (ADMA), ergothioneine (ERG), and phenylacetylglutamine, showed differential abundance. MePYR-S1, DHRSV-G2, and ERG were higher in cL than in cH, whereas ADMA and polyalkylene glycol (PAG) were elevated in cH.

### Effect of the dietary intervention on the studied markers

Previous work demonstrated the effects of a PR-diet providing ~1,391 mg/day of polyphenols (*vs.* 812 mg/day in CT), through foods such as blood orange, pomegranate juice, green tea, Renetta apple purée, dark chocolate, and berries on inflammatory markers and clinical parameters in adults aged ≥ 60 years^[20]^. Here, the influence of the PR-diet was specifically assessed in the cH cluster. Multiple variables—including physiological parameters, serum, urine, and fecal metabolites, as well as fecal microbiome composition—were analyzed using repeated measures ANOVA.

**Figure 6:**
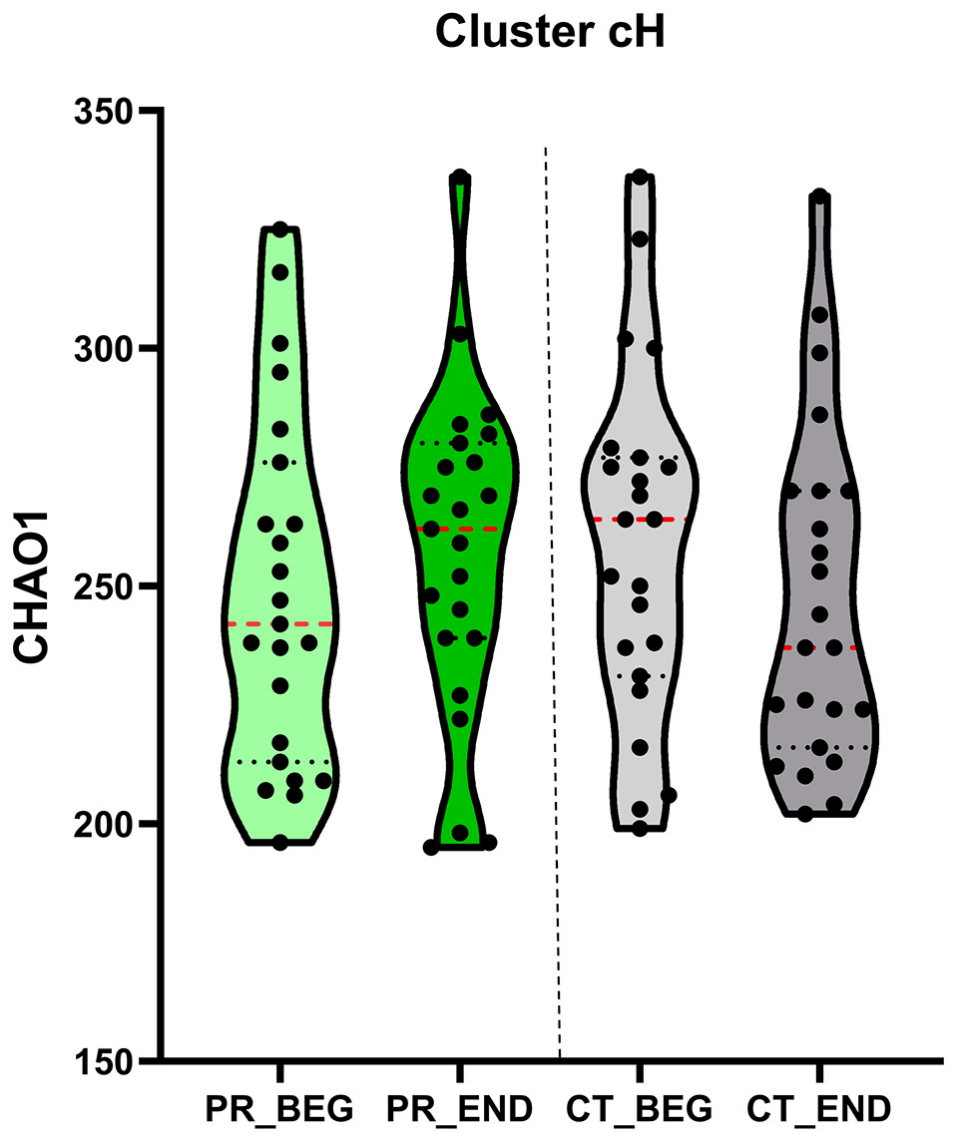
Alpha diversity. A Violin plot illustrates the results of the non-parametric repeated-measures ANOVA (ANOVA-RM) analysis of alpha diversity (Chao1 index) over the treatment period. Alpha-diversity indices are presented, with median values highlighted in red for each time point. BEG: Baseline; END: end; CT: control diet; PR: polyphenols-rich diet; ANOVA: analysis of variance; ANOVA-RM: repeated measures analysis of variance.

The PR-diet significantly reduced triglycerides (*P* = 0.01), aspartate aminotransferase (AST, *P* = 0.01), zonulin (*P* = 0.07), and serum calprotectin (*P* = 0.05) in cH subjects, while increasing LDL cholesterol (*P* = 0.05). The PR-diet also increased fecal microbiome alpha diversity (Chao1 index, *P* < 0.01) in cH subjects but not in cL [Figure 6]. Taxonomic shifts included decreases in *Methanobrevibacter smithii* (*P* = 0.06) and increases in *Blautia* spp. (*P* = 0.04), *Dorea* spp. (*P* = 0.05), and species *Eubacterium hallii* (*P* = 0.04), *Anaerostipes hadrus* (*P* = 0.01), *Blautia obeum* (*P* = 0.08), *Ruminococcus gnavus* (*P* = 0.03), and *Dorea formicigenerans* (*P* = 0.01) [Table 4]. No significant bacterial changes were observed in the cL cluster.

**Table 4:** Non-parametric ANOVA-RM of bacterial levels during treatment in the cH cluster

**cH cluster**	**NPANOVA**	**BEG**	**END**	**BEG**	**END**
**Taxonomy**	***P*-value**	**CT-diet**		**PR-diet**	
k__Archaea.p__Euryarchaeota.c__Methanobacteria.o__Methanobacteriales.f__Methanobacteriaceae.g__Methanobrevibacter.s__Methanobrevibacter_smithii	0.061	0	0	0.15	0
k__Bacteria.p__Bacteroidetes.c__Bacteroidia.o__Bacteroidales.f__Rikenellaceae.g__Alistipes.s__Alistipes_finegoldii	0.092	0	0.07	0.01	0.01
k__Bacteria.p__Firmicutes.c__Clostridia.o__Clostridiales.f__Eubacteriaceae.g__Eubacterium.s__Eubacterium_hallii	0.039	0.22	0.17	0.18	0.3
k__Bacteria.p__Firmicutes.c__Clostridia.o__Clostridiales.f__Lachnospiraceae.g__Anaerostipes.s__Anaerostipes_hadrus	0.007	0.24	0.33	0.27	0.63
k__Bacteria.p__Firmicutes.c__Clostridia.o__Clostridiales.f__Lachnospiraceae.g__Blautia	0.04	2.04	2.45	1.66	2.67
k__Bacteria.p__Firmicutes.c__Clostridia.o__Clostridiales.f__Lachnospiraceae.g__Blautia.s__Blautia_obeum	0.078	0.9	0.54	0.53	0.85
k__Bacteria.p__Firmicutes.c__Clostridia.o__Clostridiales.f__Lachnospiraceae.g__Blautia.s__Ruminococcus_gnavus	0.036	0.05	0.04	0.06	0.07
k__Bacteria.p__Firmicutes.c__Clostridia.o__Clostridiales.f__Lachnospiraceae.g__Dorea	0.054	1.03	0.66	0.74	0.89
k__Bacteria.p__Firmicutes.c__Clostridia.o__Clostridiales.f__Lachnospiraceae.g__Dorea.s__Dorea_formicigenerans	0.008	0.21	0.13	0.16	0.2
k__Bacteria.p__Firmicutes.c__Erysipelotrichia.o__Erysipelotrichales.f__Erysipelotrichaceae	0.077	0.05	0.17	0.03	0.04
k__Bacteria.p__Firmicutes.c__Firmicutes_unclassified.o__Firmicutes_unclassified.f__Firmicutes_unclassified.g__Firmicutes_unclassified.s__Firmicutes_bacterium_CAG_83	0.038	0.15	0.23	0.2	0.12

Median values for each time point are presented for the cH cluster. BEG: Baseline; END: end; CT: control diet; PR: polyphenols-rich diet; cH: higher inflammation; NPANOVA: non-parametric analysis of variance; ANOVA-RM: repeated measures analysis of variance.

Moreover, the PR-diet modulated levels of several metabolites in serum [ADMA (*P* = 0.01), PAG (*P* = 0.06), xanthosine (*P* = 0.07), oleoyl-L-carnitine, 3-methyl-2-oxovaleric acid (*P* = 0.01), ERG (*P* = 0.03), and ascorbic acid (*P* = 0.001)), urine (MePYR-S1 (*P* = 0.03), DHRSV-G2 (*P* = 0.07), 2-hydroxybutanoic acid (2-HBA, *P* = 0.01), 3′-*O*-methyl epicatechin (MeEC, *P* = 0.01), N-acetyl-S-allylcysteine (NA-SAC, *P* = 0.04)), and feces (tryptophan (*P* = 0.06)]—all of which differed between clusters (*P* < 0.1; Table 5). None of these metabolites changed significantly in the cL cluster.

**Table 5:** Repeated-measures ANOVA assessing the effect of the cH cluster across different time points within each diet (CT-diet and PR-diet)

**Cluster**	**Marker**	***P*-value**	**CT-diet BEG**	**CT-diet END**	**PR-diet BEG**	**PR-diet END**	**Higher abundance in cluster**
Physiological markers	Zonulin	0.077	39.73	44.86	39.64	38.9	cL
	Calprotectin	0.056	792.3	775.6	1,017	935.9	cH
Fecal metabolites	Tryptophan	0.06	0.1	0.09	-0.12	0.59	cL
Urinary metabolites	2-HBA	0.011	-0.18	-0.1	-0.12	0.12	cH
	MePYR-S1	0.03	-0.12	-0.19	-0.3	0.12	cL
	MeEC-S6	0.003	-0.12	-0.24	-0.02	0.56	cH
	DHRSV-G2	0.069	-0.28	-0.23	-0.23	-0.06	cL
	NA-SAC	0.044	-0.08	-0.15	-0.2	-0.26	cL
	PAG	0.062	0.28	0.07	0.22	0.18	cH
	ADMA	0.008	0.04	0.02	0.18	0.02	cH
Serum metabolites	Xanthosine	0.075	-0.18	-0.21	-0.13	0.11	cH
	Oleoylcarnitine	0.026	-0.12	-0.09	-0.13	0.06	cH
	ERG	0.026	-0.37	-0.34	-0.2	0.2	cL
	4-3-methyl-2-oxovaleric acid	0.046	-0.07	-0.05	-0.16	-0.04	cL
	Ascorbic acid	0.031	-0.06	-0.13	-0.15	-0.09	cL

For each physiological marker and metabolite (from feces, urine, and serum), the *P*-value reflects the main effect of cluster. Columns BEG and END present mean values at the beginning and end of the intervention for each diet. The final column indicates the cluster (cH or cL) in which the variable shows higher abundance. BEG: Baseline; END: end; CT: control diet; PR: polyphenols-rich diet; ANOVA: analysis of variance; cH: higher inflammation; cL: lower inflammation; 2-HBA: 2-hydroxybutanoic acid; MePYR-S1: 2-methylpyrogallol sulfate; MeEC-S6: methyl(epi)catechin sulfate 6; DHRSV-G2: dihydroresveratrol; NA-SAC: N-acetyl-S-allylcysteine; PAG: polyalkylene glycol; ERG: ergothioneine.

### Correlation between microbiome and metabolites affected by treatment

Correlation analyses at baseline examined relationships among physiological markers, microbiome taxa, and metabolites, focusing on variables altered by the dietary intervention and differing between clusters. A negative correlation was observed between at least one inflammatory marker and all bacteria that changed post-treatment (highlighted in bold in Supplementary Figure 1), except for *Ruminococcus gnavus*.

*A*. *halii* and *A. hadrus* showed positive correlations with zonulin and ERG, and negative correlations with CRP and 3-methyl-2-oxovaleric acid. *Blautia* spp. and *B*. *obeum* were negatively correlated with sICAM-1, sVCAM-1, TNF-α, and positively correlated with urine MePYR-S1. *Dorea* spp. and *D. formicigenerans* exhibited positive and negative correlations with diastolic blood pressure (DBP) and IL-6, respectively.

Fecal tryptophan negatively correlated with *Methanobrevibacter smithii*, while serum phenylacetylglutamine and ADMA positively correlated with this microorganism. *A. halii* was negatively correlated with these two metabolites, while *Blautia* spp. and *Ruminococcus gnavus* positively correlated with tryptophan.

**Figure 7:**
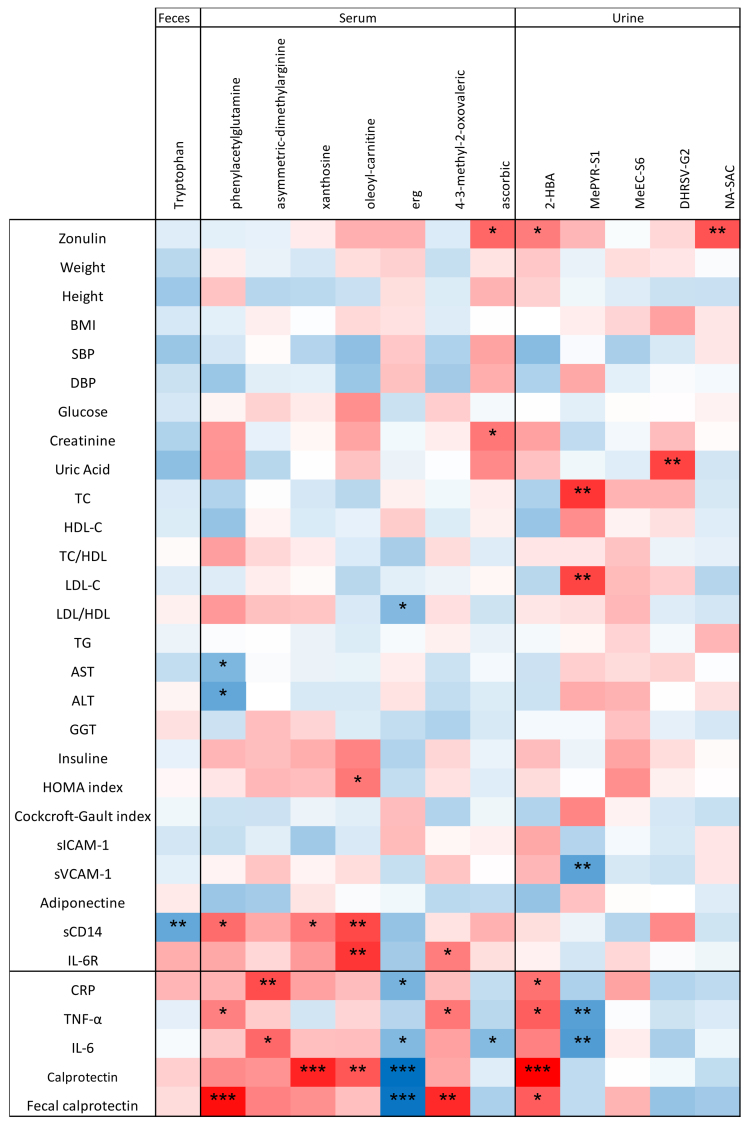
Correlation between physiological parameters/inflammatory markers and metabolites that significantly changed after intervention. The Kendall test was used to assess correlations. The ꞇ index defines the color scale (blue for negative correlation, red for positive correlation). Asterisks indicate significance levels: ^*^0.1 < *P* < 0.05; ^**^0.001 < *P* < 0.01; ^***^P < 0.001.

To provide a comprehensive overview, Kendall’s tau test was used to investigate correlations between the physiological /inflammatory markers and metabolites significantly altered by the PR-diet [Figure 7]. Negative correlations were found between several inflammatory markers and the serum metabolite ERG and urinary MePYR-S1. In contrast, PAG, ADMA, xanthosine, oleoyl-L-carnitine, 4-3-methyl-2-oxovaleric acid, and 2-HBA showed positive correlations with inflammatory markers [Figure 7].

## DISCUSSION

Given the post-hoc nature of the analyses in this study, all observed relationships between dietary intervention, inflammatory markers, gut microbiota, and metabolome should be interpreted as correlational rather than indicative of causality. This study stratified individuals aged ≥ 60 years participating in the MaPLE trial into two groups based on inflammatory markers. Specifically, 23 subjects with high inflammation (the cH group), exhibiting significantly higher levels of CRP, TNF-α, IL-6, calprotectin, and fecal calprotectin, were compared to the cL group. The average CRP concentration in the cH group exceeded 10 mg/L^[25]^, indicating active inflammation, whereas mean values in the cL group were below this threshold, suggesting the absence of high-grade inflammation. A similar pattern was observed for IL-6, with the cH cluster showing higher mean concentrations (5.19 pg/mL) in healthy individuals^[26]^. For TNF-α and both serum and fecal calprotectin, although cH cluster values were higher than those of the cL cluster, they remained within the normal range for healthy adults aged ≥ 60 years^[25,27,28]^.

Compared to our earlier MaPLE trial publication, which reported the overall effects of a PR-diet on inflammatory and clinical parameters in adults aged ≥ 60 years, this post-hoc analysis introduces a novel perspective by stratifying participants based on their baseline inflammatory profile. This approach enabled the identification of subgroup-specific responses at the microbiome and metabolomic levels, revealing that subjects with higher systemic inflammation exhibited distinct microbial and metabolic shifts following the PR-diet intervention. These findings highlight the potential for personalized nutritional strategies targeting inflammaging.

Inflammaging is characterized by chronically elevated plasma concentrations of pro-inflammatory cytokines, including IL-6 and TNF-α, along with secondary increases in markers such as CRP^[29]^. Both IL-6 and TNF-α are key signaling molecules released during macrophage activation; elevated concentrations indicate an inflammatory response triggered by infection detection^[30]^. High serum IL-6 levels can predict disability onset and are inversely associated with leg and handgrip strength in individuals aged ≥ 60 years^[31]^. CRP, a pentameric protein synthesized by the liver in response to IL-6 during inflammation^[32]^, has been linked to vascular disease, respiratory issues, cancer, and increased mortality risk^[33]^. Notably, a composite inflammatory index including CRP, IL-6, and TNF-α has been shown to predict all-cause mortality in adults aged ≥ 60 years^[34]^. Calprotectin is a soluble protein released rapidly from white cells during local inflammation or infection. Fecal calprotectin serves as a marker of intestinal inflammation, used in screening for IBD and monitoring mucosal healing or relapse^[35]^, while serum calprotectin may more accurately reflect systemic inflammation^[36]^.

Analysis of the microbiome in individuals aged ≥ 60 years revealed reduced diversity and decreased abundance of beneficial bacteria such as *Bifidobacterium* spp., *Lactobacillus* spp.^[29]^, and *Lachnospiraceae* spp.^[37]^. Salazar *et al.* demonstrated that dietary interventions restoring these bacteria can reduce pro-inflammatory cytokines and plasma CRP levels, implying that manipulating the GM may influence the function of the aging immune system^[38]^.

Our trial found microbiome compositional differences aligned with inflammatory stratification. Specifically, members of the Clostridiales order were less abundant in the cH group compared to cL. Families such as Lachnospiraceae, Eubacteriaceae, Ruminococcaceae, genera including *Dorea*, *Ruminococcus*, *Eubacterium*, and *Anaerostipes*, and species such as *Anaerobutyricum hallii*, *Anaerostipes hadrus* and *Ruminococcus bromii* were lower in cH but increased following the PR-diet. These taxa are butyrate producers implicated in maintaining intestinal homeostasis. Biagi *et al.*, in their work on the GM and inflammatory status in seniors and centenarians, reported inverse correlations between members of *Clostridium* cluster XIVa (e.g., *Eubacterium*, *Ruminococcus*, *Dorea*) and systemic inflammation^[39]^, suggesting that these taxa may serve as microbiome markers of longevity in Chinese and Italian centenarians^[39]^. Similarly, Lachnospiraceae, Ruminococcaceae, and the genus *Blautia* have been proposed as indicators of healthy longevity^[40]^. Seishima *et al.* found consistent depletion of *Dorea* spp., unclassified *Lachnospiraceae*, *Ruminococcus* spp., and *A. halii* in IBD and acute colitis patients, highlighting their potential role in intestinal homeostasis^[41]^.

In the MaPLE trial, the PR-diet involved an average threefold increase in polyphenol intake compared to the CT-diet^[20]^. Plant polyphenols are recognized anti-inflammatory agents with antimicrobial and prebiotic properties^[20]^. Their ability to modulate gut microbiota composition may contribute to their anti-inflammatory effects^[18]^. Here, the PR-diet enhanced alpha-diversity richness in the cH group and increased *Blautia* spp. and *Blautia obeum*. This increase primarily drove the elevation of the thiazole biosynthesis pathway after the PR-diet, with thiazole derivatives known for broad pharmacological activities, including anti-inflammatory, antiallergic, antibacterial, antitumor, and antihyperlipidemic effects^[18]^. We speculate that these microbiome changes contributed to observed reductions in serum calprotectin^[18]^ and zonulin (a marker of IP)^[20]^ in the cH group. Interestingly, zonulin levels were unexpectedly lower in the cH compared to the cL at baseline, but decreased further after intervention only in the cH group.

Overall, clustering adults aged ≥ 60 years based on inflammatory markers corresponded with distinct fecal microbiota compositions, supporting a strong link between inflammaging and gut microbiota. The PR-diet increased alpha diversity and expanded Clostridiales members, which are associated with gut health and homeostasis. It also modulated physiological markers (calprotectin and zonulin) and serum, urine, and fecal metabolites, promoting an anti-inflammatory profile in the adults aged ≥ 60 years with higher baseline inflammation. Collectively, these findings demonstrate a significant anti-inflammatory effect of the PR-diet, including improved IP and a distinct metabolic signature in this subgroup. This advances our understanding of the complex diet-microbiome-inflammation interplay in aging and informs dietary strategies for healthy aging.

Additional metabolites linked to inflammaging, such as 3-methyl-2-oxovaleric acid, oleoyl-carnitine, xanthosine, ADMA, and PAG, were elevated in the cH group. After the PR-diet, levels of 3-methyl-2-oxovaleric acid and oleoyl-carnitine decreased; these compounds are typically increased in maple syrup urine disease, a metabolic disorder caused by branched-chain alpha-keto acid dehydrogenase deficiency^[20]^. Fujiwara *et al.*^[42]^ reported oleoyl-carnitine acts as an oncometabolite by activating signal transducers associated with stem cell properties, potentially contributing to non-alcoholic fatty liver disease pathophysiology^[18]^. We also observed a reduction in ADMA post-intervention; elevated ADMA, an endogenous nitric oxide synthase inhibitor, is linked to cardiovascular diseases^[18]^ and interacts with CRP to influence cardiovascular events^[18]^. This aligns with the strong correlation we found between CRP and ADMA in adults aged ≥ 60 years.

Regarding cardiovascular risk, Ottosson *et al.*^[43]^ identified PAG as a GM-derived plasma metabolite associated with future coronary artery disease risk^[18]^. In our study, TNF-α and fecal calprotectin positively correlated with PAG, whose serum levels decreased following the PR-diet.

Several urinary metabolites derived from gut microbial metabolism, including 2-hydroxybenzoic acid (2-HBA), 1-O-methylpyrogallol, and dihydroresveratrol, showed significant changes after the PR-diet. Notably, 2-HBA positively correlated with all inflammatory markers, including IL-6, contradicting previous reports where this non-flavonoid polyphenol was shown to play a role in modulating both inflammation and cancer proliferation. In fact, 2-HBA was reported to activate the transcription factor NF-κB, which binds promoter regions of genes such as COX-2, suppressing inflammation and proliferation while promoting apoptosis^[44]^. We observed an increase in 2-HBA after the PR-diet, aligning with the hypothesis about the positive effects of the PR-diet on adults aged ≥ 60 years with higher levels of inflammatory markers.

The bacterial lignin-derived metabolite 1-O-methylpyrogallol was more abundant in the cL group and negatively correlated with TNF-α and IL-6, suggesting anti-inflammatory properties. Its increase after the PR-diet indicates a beneficial effect in subjects with higher inflammation.

Resveratrol is metabolized by gut bacteria into stilbene metabolites, including dihydroresveratrol (DHRSV) and lunularin (LUN), as well as 3-(4-hydroxyphenyl)-propionic acid. This microbial metabolism enhances resveratrol’s bioavailability and bioactivity. Zhang *et al.* reported that DHRSV and 3-(4-hydroxyphenyl)-propionic acid improve intestinal barrier function and mitigate gut inflammation both *in vitro* and *in vivo*^[45]^. A recent study noted that while resveratrol improves insulin sensitivity, its metabolite DHRSV has limited effects on inflammatory pathways^[46]^. This may explain the lack of correlation between inflammatory markers and DHRSV-G2 in our cohort. Interestingly, DHRSV-G2 was more abundant in the cH group than in the cL at baseline and increased further following the PR-diet in the cH participants. The PR-diet’s polyphenol-rich foods—dark chocolate, berries, and green tea—are major sources of flavan-3-ols, anthocyanins, and catechins, respectively, likely precursors of metabolites such as DHRSV-G2. These compounds may also modulate gut taxa such as *Blautia* and *Dorea*, which responded differently to the PR-diet versus control. Although methyl(epi)catechin sulfate did not correlate with inflammatory markers, it increased significantly after the PR-diet. This metabolite arises via gut microbiota methylation of epicatechin^[47]^, a phytochemical compound abundant in cocoa, berries, and other MaPLE foods^[48]^. Epicatechin protects the mucosa by reducing oxidative stress and preserving glutathione^[49]^. Its anti-inflammatory effects include lowering COX-2 levels and promoting epithelial repair via epidermal growth factor (EGF) expression^[49,50]^.

Ascorbic acid also positively modulates inflammaging and immunosenescence by scavenging free radicals and suppressing pro-inflammatory gene expression, exerting neuroprotective effects against neuroinflammation^[51]^. In our data, serum ascorbic acid increased significantly post-treatment and inversely correlated with IL-6.

These results provide a foundation for future intervention studies exploring dietary polyphenols in modulating age-related inflammation. Dose-response trials are necessary to determine optimal intake levels and intervention durations for meaningful immunometabolic benefits. Parallel investigations of specific polyphenol subclasses—such as flavan-3-ols, anthocyanins, and stilbenes—could identify the most bioactive compounds or food sources. Integrating microbiome and metabolome profiling into nutritional research may enable personalized dietary strategies tailored to individuals’ inflammatory status and gut microbial signatures, enhancing the efficacy of interventions targeting inflammaging.

## LIMITATIONS

This study has several limitations that should be carefully considered when interpreting the findings. First, as a post-hoc analysis, it is inherently limited in establishing causal relationships. Although the original intervention was a randomized controlled trial, the post-hoc nature introduces potential uncontrolled confounders and biases not accounted for in the original design. Therefore, observed associations should be viewed as exploratory and hypothesis-generating rather than conclusive evidence of causality.

Second, generalizability is limited by the study population characteristics. The participants were Italian adults aged ≥ 60 years residing in long-term care facilities, who may differ substantially from community-dwelling adults aged ≥ 60 years in health status, comorbidities, medications, physical activity, diet, and lifestyle. These factors may affect baseline gut microbiota and dietary response. Caution is advised when extrapolating these results to broader aging populations, especially across different geographic, cultural, or healthcare contexts.

Third, despite monitoring and some control of dietary intake, variability in metabolism and microbiota composition likely influenced individual responses. Adherence was assessed primarily through self-reports and staff observation, which may not fully capture day-to-day compliance or minor deviations.

Fourth, although metagenomic and metabolomic analyses of fecal samples were conducted, metatranscriptomic and proteomic data were not included. Thus, while the functional potential of the microbiota was assessed, actual gene expression and protein activity remain unknown. Incorporating metatranscriptomics would provide insights into active microbial pathways responding to the diet, and proteomics could reveal dynamic host-microbe interactions. Moreover, fecal metabolomics reflects primarily distal colon metabolism, which may not represent systemic or proximal gut microbial activity.

Fifth, although the sample size was adequate for primary outcomes, it may lack power to detect subtle microbiome and metabolome changes, especially when stratified by inflammatory status. High interindividual variability, common in adults aged ≥ 60 years, further complicates the identification of consistent patterns in subgroup analyses. This underscores the need for larger, well-powered studies to confirm these findings.

Sixth, the study lacked a long-term follow-up to assess the durability of microbial, metabolic, and inflammatory changes beyond the 8-week intervention. Thus, the persistence of benefits and the necessity of repeated or prolonged PR-diet exposure remain unknown.

Finally, despite efforts to minimize confounding, residual confounders cannot be excluded. Future studies should address these limitations by including larger, more diverse populations, extended follow-up, and comprehensive multi-omics approaches to better capture systemic and functional effects of dietary interventions.

In conclusion, this post-hoc analysis demonstrates that a PR-diet beneficially modulates systemic inflammation and gut microbiota composition in adults aged ≥ 60 years, particularly those with elevated baseline inflammation. These findings highlight the potential of targeted nutritional strategies to support healthy aging and pave the way for precision nutrition approaches that leverage specific foods, bioactive compounds, and individual microbiome profiles to counteract inflammaging and its related health burdens.

## DECLARATIONS

### Availability of data and materials

Raw sequencing data have been deposited in the European Nucleotide Archive (ENA) under the accession number PRJEB76236 and are publicly available at: https://www.ebi.ac.uk/ena/browser/view/PRJEB76236. Additional data relevant to this study are available from the corresponding authors upon reasonable request.

### Copyright

© The Author(s) 2025.
