# Supplementary material for: Effect of a polyphenol-rich dietary pattern on subjects aged ≥ 60 years with higher levels of inflammatory markers: insights into microbiome and metabolome
Source: Microbiome Res Rep. 2025 Oct 21;4(4):38. doi: 10.20517/mrr.2025.33 (PMC12702650; doi:10.20517/mrr.2025.33)
Supplement: Supplementary file 1 [file mrr-4-4-38-SupplementaryMaterials.pdf]

Supplementary Materials

Effect of a polyphenol-rich dietary pattern on subjects aged ≥ 60 years with higher levels of inflammatory markers: insights into microbiome and metabolome

Giorgio Gargari[1], Tomas Meroño[2,3], Gregorio Peron[2,4], Cristian Del Bo'[1], Mirko Marino[1], Antonio Cherubini[5,6], Cristina Andres-Lacueva[2,3], Paul Antony Kroon[7], Patrizia Riso[1], Simone Guglielmetti[8]

[1]Department of Food, Environmental and Nutritional Sciences (DeFENS), Universita Degli Studi di Milano, Milan 20133, Italy.

[2]Biomarkers and Nutrimetabolomics Laboratory, Department de Nutrició, Ciències de l'Alimentació i Gastronomia, Institut de Recerca en Nutrició i Seguretat Alimentària (INSA), Facultat de Farmàcia i Ciències de l'Alimentació, Universitat de Barcelona (UB), Barcelona 08028, Spain.

[3]Centro de Investigación Biomédica en Red de Fragilidad y Envejecimiento Saludable (CIBERFES), Instituto de Salud Carlos III, Madrid 28029, Spain.

[4]Department of Molecular and Translational Medicine, University of Brescia, Brescia 25121, Italy.

[5]Department of Geriatric Pathways of Frailty, Continuity of Care and Rehabilitation, Istituto di Ricovero e Cura a Carattere Scientifico - Istituto Nazionale di Ricovero e Cura per Anziani (IRCCS INRCA), Ancona 60127, Italy.

[6]Department of Clinical and Molecular Sciences, Università Politecnica delle Marche, Ancona 60131, Italy.

[7]Quadram Institute Bioscience, Norwich Research Park, Norwich NR4 7UQ, UK.

[8]µbEat lab, Department of Biotechnology and Biosciences (BtBs), University of Milano-Bicocca, Milan 20126, Italy.

Correspondence to: Dr. Giorgio Gargari, Department of Food, Environmental and Nutritional Sciences (DeFENS), Universita Degli Studi di Milano, Milan 20133, Italy. E-mail: giorgio.gargari@unimi.it; Prof. Simone Guglielmetti, µbEat lab, Department of Biotechnology and Biosciences (BtBs), University of Milano-Bicocca, Milano 20126, Italy. E-mail: simone.guglielmetti@unimib.it

Supplementary Table 1. Comparative analysis of metabolite levels in fecal, serum, and urine samples between clusters cL and cH

	Metabolites	p-value		Median/mean	
		Normality test	Mann-Whitney/Ttest	cH	cl
**Feces**	4-Aminobutyrate (gaba)	0.000	0.005	▼0.00	▲0.18
	Glycerol	0.000	0.030	▲0.66	▼0.33
	Nicotinate	0.000	0.008	▲0.14	▼0.11
**Serum**	2-hba	0.000	0.049	▼-0.23	▲-0.09
	3-hha	0.131	0.017	▲0.02	▼-0.37
	3-hphpa	0.442	0.013	▲0.09	▼-0.33
	3-methylindole	0.306	0.011	▲-0.01	▼-0.17
	3-methyl-2-oxovaleric	0.165	0.028	▼0.27	▲0.41
	4-imidazoleacrylic	0.360	0.044	▼-0.04	▲0.23
	asymmetric-dimethylarginine	0.028	0.015	▼-0.12	▲0.08
	betaine	0.122	0.030	▲0.13	▼-0.12
	dheas	0.814	0.020	▲0.24	▼-0.14
	dodecanedioic	0.006	0.006	▲-0.08	▼-0.27
	erg	0.462	0.002	▲0.27	▼-0.26
	hexanoic	0.010	0.036	▲-0.07	▼-0.20
	indolepropionic	0.000	0.039	▲0.07	▼-0.25
	n-acetylglucosamine-6s	0.003	0.031	▼-0.13	▲0.07
	pentadecanoic	0.000	0.002	▼-0.13	▲0.24
	phenylacetylglutamine	0.002	0.036	▼0.05	▲0.24
	pimelic	0.381	0.033	▲0.07	▼-0.21
	propionic	0.000	0.042	▲-0.05	▼-0.12
	riboflavin	0.023	0.025	▼-0.29	▲-0.05
	tryptophan	0.211	0.016	▲0.11	▼-0.04
	vanillylmandelic	0.689	0.007	▼-0.22	▲0.09
	xanthurenic	0.515	0.006	▲0.14	▼-0.06
**Urine**	4-HBAld	0.048	0.020	▼-0.39	▲-0.03
	DHRSV-G2	0.000	0.041	▲-0.08	▼-0.28
	ED-S	0.569	0.010	▲0.14	▼-0.32
	FA	0.182	0.008	▼-0.14	▲0.44
	FOLIN	0.706	0.010	▼-0.19	▲0.10
	iVA	0.518	0.035	▼-0.23	▲0.16
	MeGA-S2	0.511	0.045	▲0.21	▼-0.26
	MePYR-S1	0.787	0.020	▲0.12	▼-0.38
	oCOU-G	0.102	0.037	▲0.14	▼-0.27
	TYR-S	0.000	0.019	▲-0.19	▼-0.26
	VAN-S	0.061	0.039	▼-0.33	▲0.19

Only metabolites with $P \leq 0.05$ are included. *P*-values were calculated using both parametric (*t*-test) and non-parametric (Mann-Whitney *U*) tests, according to the results of the Shapiro-Francia normality test. Mean or median values are reported depending on the applied test.

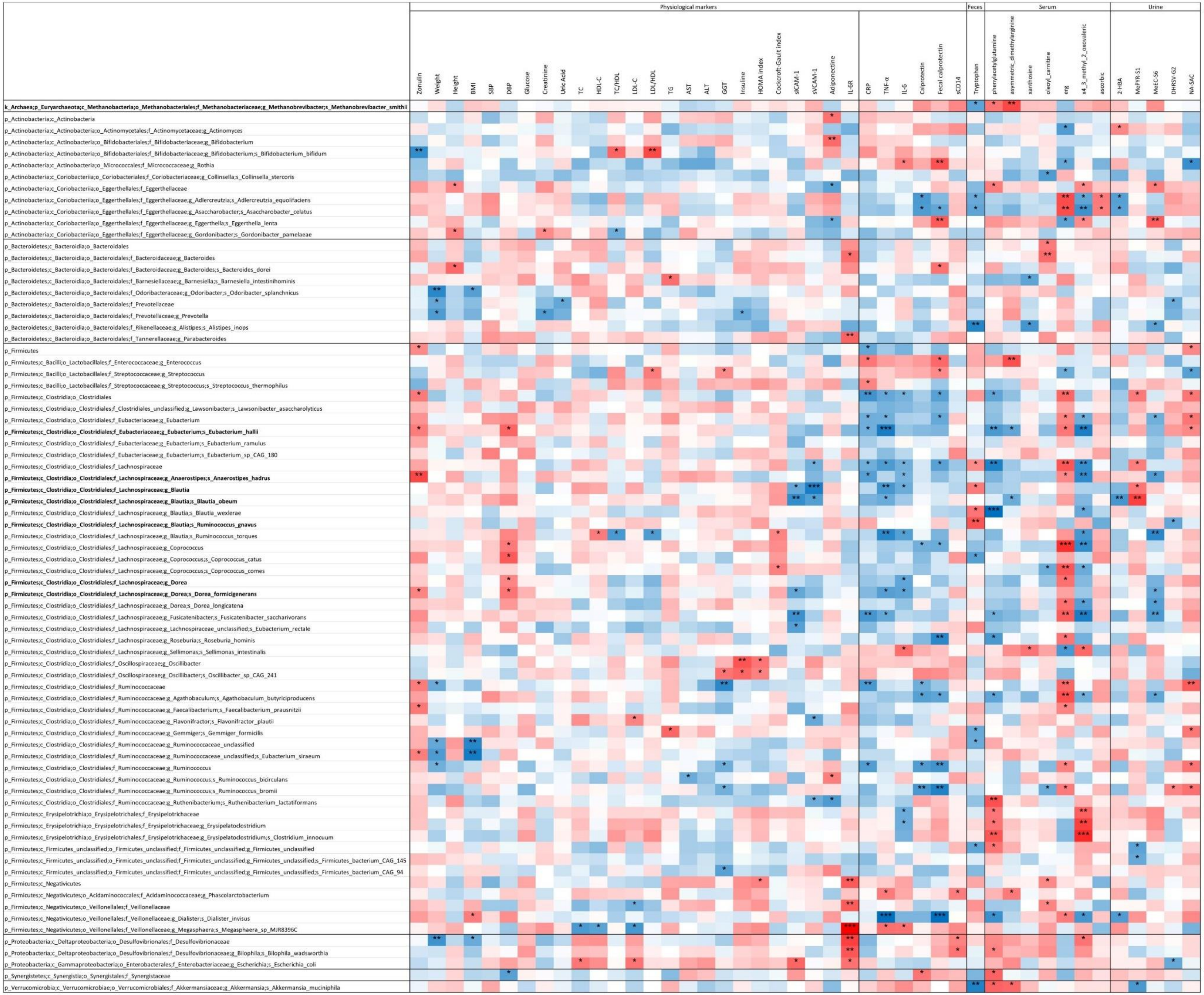

Physiological markers
Feces
Serum
Urine
Zonulin
Weight
Height
BMI
SBP
DBP
Glucose
Creatinine
Uric Acid
TC
HDL-C
TC/HDL
LDL-C
LDL/HDL
TG
AST
ALT
GGT
Insuline
HOMA index
Cockcroft-Gault index
sICAM-1
sVCAM-1
Adiponectine
IL-6R
CRP
TNF-α
IL-6
Calprotectin
Fecal calprotectin
sCD14
Tryptophan
phenylacetylglutamine
asymmetric_dimethylarginine
xanthosine
oleoyl_carnitine
erg
x4_3_methyl_2_oxovaleric
ascorbic
2-HBA
MePYR-S1
MeEC-S6
DHRSV-G2
NA-SAC
k_Archaea;p_Euryarchaeota;c_Methanobacteria;o_Methanobacteriales;f_Methanobacteriaceae;g_Methanobrevibacter;s_Methanobrevibacter_smithii
p_Actinobacteria;c_Actinobacteria
p_Actinobacteria;c_Actinobacteria;o_Actinomycetales;f_Actinomycetaceae;g_Actinomyces
p_Actinobacteria;c_Actinobacteria;o_Bifidobacteriales;f_Bifidobacteriaceae;g_Bifidobacterium
p_Actinobacteria;c_Actinobacteria;o_Bifidobacteriales;f_Bifidobacteriaceae;g_Bifidobacterium;s_Bifidobacterium_bifidum
p_Actinobacteria;c_Actinobacteria;o_Micrococcales;f_Micrococcaceae;g_Rothia
p_Actinobacteria;c_Coriobacteriia;o_Coriobacteriales;f_Coriobacteriaceae;g_Collinsella;s_Collinsella_stercoris
p_Actinobacteria;c_Coriobacteriia;o_Eggerthellales;f_Eggerthellaceae
p_Actinobacteria;c_Coriobacteriia;o_Eggerthellales;f_Eggerthellaceae;g_Adlercreutzia;s_Adlercreutzia_equolifaciens
p_Actinobacteria;c_Coriobacteriia;o_Eggerthellales;f_Eggerthellaceae;g_Asaccharobacter;s_Asaccharobacter_celatus
p_Actinobacteria;c_Coriobacteriia;o_Eggerthellales;f_Eggerthellaceae;g_Eggerthella;s_Eggerthella_lenta
p_Actinobacteria;c_Coriobacteriia;o_Eggerthellales;f_Eggerthellaceae;g_Gordonibacter;s_Gordonibacter_pamelaeae
p_Bacteroidetes;c_Bacteroidia;o_Bacteroidales
p_Bacteroidetes;c_Bacteroidia;o_Bacteroidales;f_Bacteroidaceae;g_Bacteroides
p_Bacteroidetes;c_Bacteroidia;o_Bacteroidales;f_Bacteroidaceae;g_Bacteroides;s_Bacteroides_dorei
p_Bacteroidetes;c_Bacteroidia;o_Bacteroidales;f_Barnesiellaceae;g_Barnesiella;s_Barnesiella_intestinihominis
p_Bacteroidetes;c_Bacteroidia;o_Bacteroidales;f_Odoribacteraceae;g_Odoribacter;s_Odoribacter_splanchnicus
p_Bacteroidetes;c_Bacteroidia;o_Bacteroidales;f_Prevotellaceae
p_Bacteroidetes;c_Bacteroidia;o_Bacteroidales;f_Prevotellaceae;g_Prevotella
p_Bacteroidetes;c_Bacteroidia;o_Bacteroidales;f_Rikenellaceae;g_Alistipes;s_Alistipes_inops
p_Bacteroidetes;c_Bacteroidia;o_Bacteroidales;f_Tannerellaceae;g_Parabacteroides
p_Firmicutes
p_Firmicutes;c_Bacilli;o_Lactobacillales;f_Enterococcaceae;g_Enterococcus
p_Firmicutes;c_Bacilli;o_Lactobacillales;f_Streptococcaceae;g_Streptococcus
p_Firmicutes;c_Bacilli;o_Lactobacillales;f_Streptococcaceae;g_Streptococcus;s_Streptococcus_thermophilus
p_Firmicutes;c_Clostridia;o_Clostridiales
p_Firmicutes;c_Clostridia;o_Clostridiales;f_Clostridiales_unclassified;g_Lawsonibacter;s_Lawsonibacter_asaccharolyticus
p_Firmicutes;c_Clostridia;o_Clostridiales;f_Eubacteriaceae;g_Eubacterium
p_Firmicutes;c_Clostridia;o_Clostridiales;f_Eubacteriaceae;g_Eubacterium;s_Eubacterium_hallii
p_Firmicutes;c_Clostridia;o_Clostridiales;f_Eubacteriaceae;g_Eubacterium;s_Eubacterium_ramulus
p_Firmicutes;c_Clostridia;o_Clostridiales;f_Eubacteriaceae;g_Eubacterium;s_Eubacterium_sp_CAG_180
p_Firmicutes;c_Clostridia;o_Clostridiales;f_Lachnospiraceae
p_Firmicutes;c_Clostridia;o_Clostridiales;f_Lachnospiraceae;g_Anaerostipes;s_Anaerostipes_hadrus
p_Firmicutes;c_Clostridia;o_Clostridiales;f_Lachnospiraceae;g_Blautia
p_Firmicutes;c_Clostridia;o_Clostridiales;f_Lachnospiraceae;g_Blautia;s_Blautia_obeum
p_Firmicutes;c_Clostridia;o_Clostridiales;f_Lachnospiraceae;g_Blautia;s_Blautia_wexlerae
p_Firmicutes;c_Clostridia;o_Clostridiales;f_Lachnospiraceae;g_Blautia;s_Ruminococcus_gnavus
p_Firmicutes;c_Clostridia;o_Clostridiales;f_Lachnospiraceae;g_Blautia;s_Ruminococcus_torques
p_Firmicutes;c_Clostridia;o_Clostridiales;f_Lachnospiraceae;g_Coprococcus
p_Firmicutes;c_Clostridia;o_Clostridiales;f_Lachnospiraceae;g_Coprococcus;s_Coprococcus_catus
p_Firmicutes;c_Clostridia;o_Clostridiales;f_Lachnospiraceae;g_Coprococcus;s_Coprococcus_comes
p_Firmicutes;c_Clostridia;o_Clostridiales;f_Lachnospiraceae;g_Dorea
p_Firmicutes;c_Clostridia;o_Clostridiales;f_Lachnospiraceae;g_Dorea;s_Dorea_formicigenerans
p_Firmicutes;c_Clostridia;o_Clostridiales;f_Lachnospiraceae;g_Dorea;s_Dorea_longicatena
p_Firmicutes;c_Clostridia;o_Clostridiales;f_Lachnospiraceae;g_Fusicatenibacter;s_Fusicatenibacter_saccharivorans
p_Firmicutes;c_Clostridia;o_Clostridiales;f_Lachnospiraceae;g_Lachnospiraceae_unclassified;s_Eubacterium_rectale
p_Firmicutes;c_Clostridia;o_Clostridiales;f_Lachnospiraceae;g_Roseburia;s_Roseburia_hominis
p_Firmicutes;c_Clostridia;o_Clostridiales;f_Lachnospiraceae;g_Sellimonas;s_Sellimonas_intestinalis
p_Firmicutes;c_Clostridia;o_Clostridiales;f_Oscillospiraceae;g_Oscillibacter
p_Firmicutes;c_Clostridia;o_Clostridiales;f_Oscillospiraceae;g_Oscillibacter;s_Oscillibacter_sp_CAG_241
p_Firmicutes;c_Clostridia;o_Clostridiales;f_Ruminococcaceae
p_Firmicutes;c_Clostridia;o_Clostridiales;f_Ruminococcaceae;g_Agathobaculum;s_Agathobaculum_butyriciproducens
p_Firmicutes;c_Clostridia;o_Clostridiales;f_Ruminococcaceae;g_Faecalibacterium;s_Faecalibacterium_prausnitzii
p_Firmicutes;c_Clostridia;o_Clostridiales;f_Ruminococcaceae;g_Flavonifractor;s_Flavonifractor_plautii
p_Firmicutes;c_Clostridia;o_Clostridiales;f_Ruminococcaceae;g_Gemmiger;s_Gemmiger_formicilis
p_Firmicutes;c_Clostridia;o_Clostridiales;f_Ruminococcaceae;g_Ruminococcaceae_unclassified
p_Firmicutes;c_Clostridia;o_Clostridiales;f_Ruminococcaceae;g_Ruminococcaceae_unclassified;s_Eubacterium_siraeum
p_Firmicutes;c_Clostridia;o_Clostridiales;f_Ruminococcaceae;g_Ruminococcus
p_Firmicutes;c_Clostridia;o_Clostridiales;f_Ruminococcaceae;g_Ruminococcus;s_Ruminococcus_bicirculans
p_Firmicutes;c_Clostridia;o_Clostridiales;f_Ruminococcaceae;g_Ruminococcus;s_Ruminococcus_bromii
p_Firmicutes;c_Clostridia;o_Clostridiales;f_Ruminococcaceae;g_Ruthenibacterium;s_Ruthenibacterium_lactatiformans
p_Firmicutes;c_Erysipelotrichia;o_Erysipelotrichales;f_Erysipelotrichaceae
p_Firmicutes;c_Erysipelotrichia;o_Erysipelotrichales;f_Erysipelotrichaceae;g_Erysipelatoclostridium
p_Firmicutes;c_Erysipelotrichia;o_Erysipelotrichales;f_Erysipelotrichaceae;g_Erysipelatoclostridium;s_Clostridium_innocuum
p_Firmicutes;c_Firmicutes_unclassified;o_Firmicutes_unclassified;f_Firmicutes_unclassified;g_Firmicutes_unclassified
p_Firmicutes;c_Firmicutes_unclassified;o_Firmicutes_unclassified;f_Firmicutes_unclassified;g_Firmicutes_unclassified;s_Firmicutes_bacterium_CAG_145
p_Firmicutes;c_Firmicutes_unclassified;o_Firmicutes_unclassified;f_Firmicutes_unclassified;g_Firmicutes_unclassified;s_Firmicutes_bacterium_CAG_94
p_Firmicutes;c_Negativicutes
p_Firmicutes;c_Negativicutes;o_Acidaminococcales;f_Acidaminococcaceae;g_Phascolarctobacterium
p_Firmicutes;c_Negativicutes;o_Veillonellales;f_Veillonellaceae
p_Firmicutes;c_Negativicutes;o_Veillonellales;f_Veillonellaceae;g_Dialister;s_Dialister_invisus
p_Firmicutes;c_Negativicutes;o_Veillonellales;f_Veillonellaceae;g_Megasphaera;s_Megasphaera_sp_MJR8396C
p_Proteobacteria;c_Deltaproteobacteria;o_Desulfovibrionales;f_Desulfovibrionaceae
p_Proteobacteria;c_Deltaproteobacteria;o_Desulfovibrionales;f_Desulfovibrionaceae;g_Bilophila;s_Bilophila_wadsworthia
p_Proteobacteria;c_Gammaproteobacteria;o_Enterobacterales;f_Enterobacteriaceae;g_Escherichia;s_Escherichia_coli
p_Synergistetes;c_Synergistia;o_Synergistales;f_Synergistaceae
p_Verrucomicrobia;c_Verrucomicrobiae;o_Verrucomicrobiales;f_Akkermansiaceae;g_Akkermansia;s_Akkermansia_muciniphila

Supplementary Figure 1. Correlation between physiological parameters and bacteria assessed using the Kendall test. The τ index defines the color scale (blue for negative correlation, red for positive correlation). Asterisks indicate significance levels: $^{*}0.1 < P < 0.05$; $^{**}0.001 < P < 0.01$; $^{***}P < 0.001$. Bacterial taxa that changed after treatment are shown in bold.
